# Dynamic monitoring of cognitive impairment and prognostic recurrence risk in patients with stroke by serum HDAC3 and FOXO1: A potential diagnostic approach

**DOI:** 10.5937/jomb0-60965

**Published:** 2026-04-15

**Authors:** Xintong Liu, Lulu Wang, Bin Xiao, Zhengkun Liu, Yue Dou

**Affiliations:** 1 Changji Branch of the First Affiliated Hospital of Xinjiang Medical University, Department of Pain Rehabilitation Medicine, Changji, China; 2 Changji Branch of the First Affiliated Hospital of Xinjiang Medical University, Department of Hypertension&Cardiac Pacing Electrophysiology Science, Changji, China; 3 Changji Branch of the First Affiliated Hospital of Xinjiang Medical University, Department of Pain Rehabilitation Medicine, Changji, Xinjiang, China; 4 Changji Branch of the First Affiliated Hospital of Xinjiang Medical University, Department of Neurology, Changji, China

**Keywords:** HDAC3, FOXO1, cerebral stroke, cognitive dysfunction, diagnosis, HDAC3, FOXO1, cerebralni moždani udar, kognitivna disfunkcija, dijagnoza

## Abstract

**Background:**

To evaluate the clinical value of dynamically monitoring serum histone deacetylase 3 (HDAC3) and forkhead box protein O1 (FOXO1) levels in evaluating cognitive impairment (CI) in cerebral stroke (CS) patients, to enable early identification of high-risk individuals and guide targeted interventions.

**Methods:**

This study included 120 CS patients admitted from March, 2023 to March, 2024, with their serum HDAC3 and FOXO1 levels examined upon admission (T0) and at 2 (T1) and 24 hours (T2) following treatment. Differences in biomarker levels (CI group vs. non-CI group) were analyzed, and the predictive value of HDAC3 and FOXO1 for CI was determined. In addition, patients were followed up for 1 year prognosis, and the predictive effect of HDAC3 and FOXO1 on the prognostic recurrence risk of CS was analyzed.

**Results:**

CI occurred in 46 cases. HDAC3 expression was markedly increased in CI cases versus non-CI patients at T0, T1, and T2, whereas FOXO1 differed significantly only at T1 and T2 (P&lt; 0.05). HDAC3 and FOXO1 showed maximal predictive value for CI at the T2 timepoint. Similarly, the increase of HDAC3 and FOXO1 was also related to the risk of prognosis recurrence of CS patients. The sensitivity and specificity of combined detection of HDAC3 and FOXO1 in predicting the prognosis recurrence of CS patients were 84.38% and 78.41% (P&lt; 0.05).

**Conclusions:**

The pattern of dynamic HDAC3 and FOXO1 changes provides a new diagnostic scheme for the development of CI and prognostic recurrence risk in CS patients.

## Abstract (sr)

**Uvod:**

Cilj je bio da se proceni klinička vrednost dinamičkog praćenja nivoa serumske histon deacetilaze 3 (HDAC3) i proteina O1 u obliku viljuškastog boksa (FOXO1) u proceni kognitivnog oštećenja (CI) kod pacijenata sa cerebralnim moždanim udarom (CS), kako bi se omogućila rana identifikacija osoba sa visokim rizikom i usmerile ciljane intervencije.

**Metode:**

Ova studija je obuhvatila 120 pacijenata sa komplementarnim poremećajima (CS) primljenih od marta 2023. do marta 2024. godine, kod kojih su im nivoi HDAC3 i FOXO1 u serumu ispitani pri prijemu (T0) i 2 (T1) i 24 sata (T2) nakon lečenja. Analizirane su razlike u nivoima biomarkera (CI grupa u odnosu na grupu bez CI) i određena je prediktivna vrednost HDAC3 i FOXO1 za CI. Pored toga, pacijenti su praćeni tokom jednogodišnje prognoze i analiziran je prediktivni efekat HDAC3 i FOXO1 na prognostički rizik od recidiva CS.

**Rezultati:**

CI se javio u 46 slučajeva. Ekspresija HDAC3 je bila značajno povećana kod CI slučajeva u odnosu na pacijente bez CI u T0, T1 i T2, dok se FOXO1 značajno razlikovao samo u T1 i T2 (P&lt; 0,05). HDAC3 i FOXO1 su pokazali maksimalnu prediktivnu vrednost za CI u vremenskoj tački T2. Slično tome, povećanje HDAC3 i FOXO1 je takođe bilo povezano sa rizikom od prognoze recidiva kod pacijenata sa CS. Osetljivost i specifičnost kombinovane detekcije HDAC3 i FOXO1 u predviđanju prognoze recidiva kod pacijenata sa CS bile su 84,38% i 78,41% (P&lt; 0,05).

**Zaključak:**

Obrazac dinamičkih promena HDAC3 i FOXO1 pruža novu dijagnostičku šemu za razvoj CI i prognostički rizik od recidiva kod pacijenata sa CS.

## Introduction

Globally, cerebral stroke (CS) ranks among the leading causes of long-term disability, leading to varying degrees of cognitive impairment (CI) in about 30%-70% of patients during the acute phase, which substantially hinders functional rehabilitation and compromises daily living standards [1]. Identifying CI early for prompt interventions has thus become a key breakthrough to enhance CS outcomes [2]. Traditional cognitive assessments are limited by insufficient timeliness, strong subjectivity, and poor early sensitivity due to their heavy dependence on neuropsychological tests and imaging techniques, impeding efficient and accurate emergency diagnostics [3]. With the rise of precision medicine, identifying biomarkers for early prediction has become a prominent research priority.

In recent years, the relationship between epigenetic regulators and neuroplasticity after brain injury has been widely concerned [4]. For instance, histone deacetylase 3 (HDAC3) regulates synaptic plasticity-associated gene expression, thereby influencing neurodegenerative disease progression [5]. Meanwhile, forkhead box protein O1 (FOXO1) regulates neuronal survival through its role in oxidative stress response and mitochondrial regulation [6]. Recently, Li N et al. [7] found that HDAC3 directly reduced FOXO1 acetylation and promoted its nuclear translocation in LPS-treated type II alveolar epithelial cells. These results suggest that HDAC3/FOXO1 signaling axis plays an important role in cell function damage [7]. This suggests that both biomarkers have important potential for CS severity assessment, though systematic research and verification are needed. Additionally, how to link the temporal changes of HDAC3 and FOXO1 with CI has yet to form a standardized evaluation system.

In this study, we will analyze how HDAC3 and FOXO1 expression affect CI in CS patients. The results will address the lack of early biomarkers for CS-induced CI, support precision time-window strategies, and advance epigenetic markers toward clinical use, with substantial academic and public health relevance.

## Materials and methods

### Research subjects

**Figure 1:**
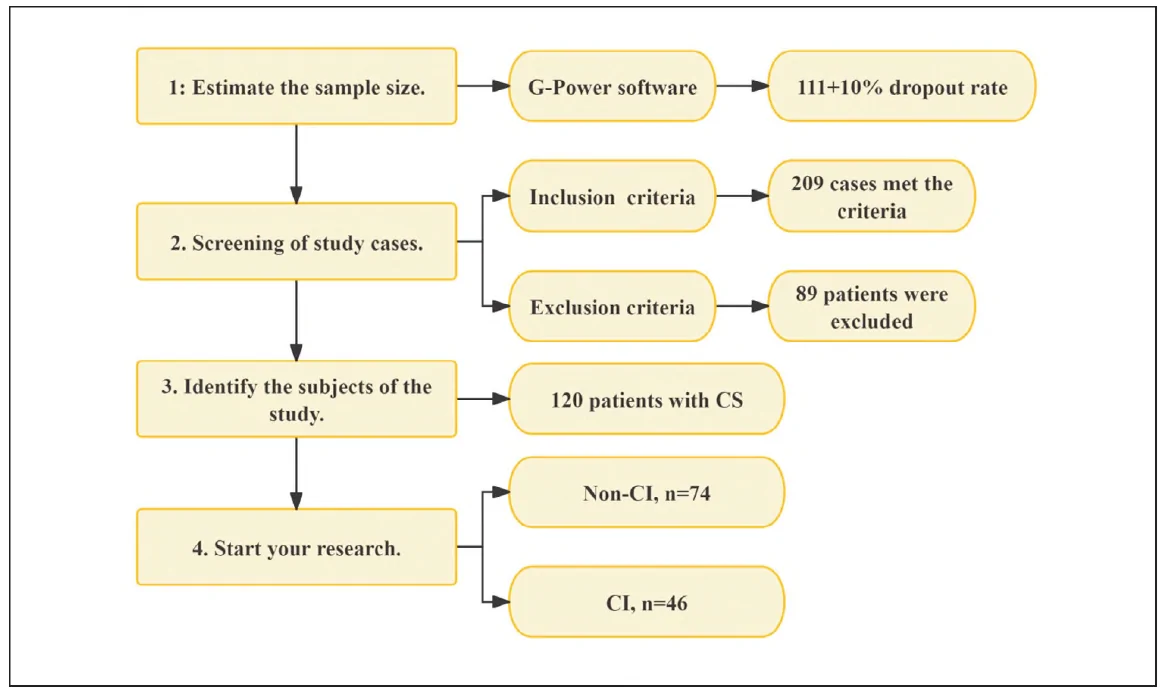
Screening of study subjects. Based on the estimation of sample size and selection of inclusion and exclusion criteria, a total of 120 CS patients were included in this study.

The study population consisted of CS patients admitted from March 2023 to March 2024. Using G*Power software (one-tailed test, effect = 0.3, α = 0.05, power=0.95, effect size=0.3 was based on pilot data showing HDAC3 Δ = 1.2 ng/mL (SD = 0.8) between CI/non-CI groups), we calculated a minimum requirement of 111 participants after considering a 10% dropout rate. After applying inclusion/ exclusion criteria, 120 eligible subjects were enrolled (10% dropout rate compensated) (detailed in Figure 1). Ethical approval was obtained from our institutional review board, and written informed consent was secured from all individuals. Patients were screened for CI using the Montreal Cognitive Assessment (MoCA) [8] within 7 days after admission, with all assessments completed within 48-72 hours to minimize temporal bias. MoCA includes 11 items in 8 cognitive fields, including attention/concentration, executive function, memory, language, visuospatial skills, abstract reasoning, calculation, and orientation. A total score below 26 (out of 30) indicates CI. The study included 120 patients, of whom 46 (38.33%) developed CI and were categorized into the CI group; the other 78 comprised the non-CI group.

### Inclusion and exclusion criteria

Inclusion criteria: Age 18-80 years with CS diagnosis [9]; acute CS on imaging (CT/MRI) within 24 hours of symptom onset; treatment with recombinant tissue plasminogen activator (rt-PA) thrombolysis or mechanical thrombectomy; National Institute of Health Stroke Scale (NIHSS) score [10] of 4-20. Exclusion criteria: Hemorrhagic CS/transient ischemic attack/subarachnoid hemorrhage; pre-existing CI. pre-existing CI (e.g., dementia) confirmed by medical records, family interviews, and baseline MoCA <18 prior to stroke onset; severe organ failure or active cancer; histone deacetylase (HDAC) inhibitor use in the past 3 months; pregnancy or breastfeeding.

### Methods

After admission, patients were quickly identified and triaged: Emergency department nurses conduct rapid CS screening using the »Stroke 1-2-0« screening scale (1: facial droop, 2: arm weakness, 0: speech impairment) within five minutes. Through direct communication with the neurology team, the triage nurse accelerated the evaluation process, skipping routine emergency department protocols. Medical staff completed both patient history documentation and NIHSS baseline scoring in under five minutes to assess thrombolysis/thrombectomy eligibility. The care team ensured completion of all necessary imaging and bloodwork within the first half-hour of hospital presentation. Rt-PA was administered for patients presenting within 4.5 hours of symptom onset without contraindications, ensuring door-to-needle time (DNT) 60 minutes. For late-presenting cases (6-24h) who met DAWN/DEFUSE 3 criteria (demonstrating salvageable ischemic penumbra), thrombectomy was performed with door-to-puncture time (DPT) ≤75 minutes. Post-thrombolysis (24 hours), daily aspirin (100 mg) + clopidogrel (75 mg) therapy was started absent hemorrhage. All patients were followed up for a period of 1 year. The follow-up was conducted by regular review, and the patients were required to have at least one recurrence per month (monthly telephone assessments + quarterly outpatient visits). The primary outcome was recurrence of CS.

### Laboratory tests

Fasting venous blood of 2 mL was collected from patients on admission (T0) and centrifuged at 3000 rpm for 10 minutes (4°C) to separate serum. Total cholesterol (TC), triglyceride (TG), low-density lipoprotein cholesterol (LDL-C), high-density lipoprotein cholesterol (HDL-C) and homocysteine (Hcy) were measured by automatic biochemical analyzer (Beckman AU5800). Optical system calibration (wavelength correction) and reaction cup cleaning were performed after the instrument was turned on. Scan the barcode of reagents, automatically identify the information of reagents (name, batch number, validity period, assigned value), and load them into the reagent warehouse in sequence. The calibrator was diluted according to the instruction manual, and the calibrator was added into the reaction cup. The instrument automatically detected and calculated the calibration coefficient (calibration curve). After the calibration passed, the quality control (at least 2 levels) was detected, and the results were recorded and compared with the target value (allowable range: ±2SD). The serum was put into the sample rack, and the instrument automatically scanned the code (or manually numbered), added the sample, and detected. The system automatically generates the results. Quality control: use third-party quality control materials with the same substrate (serum/plasma) as test samples, and the levels cover medical decision points (such as low, medium, and high values). Quality control samples (at least 2 levels) were tested daily before testing. If more than 20 samples were tested, testing was repeated every 2 hours.

At the time of T0 as well as 2 (T1) and 24 hours (T2) after treatment, 4 mL of elbow vein blood was collected for HDAC3 and FOXO1 quantification using enzyme-linked immunosorbent assay (ELISA). HDAC3: Human HDAC3 ELISA Kit (Cusabio, CSB-EL007995HU); FOXO1: Human FOXO1 ELISA Kit (Abbexa, abx253897). To be specific, the blood samples were centrifuged (3000 rpm, 10 min, 4°C) to separate serum, which was subpackaged into 1.5 mL sterile EP tubes, marked with patient ID and time point (T0/T1/T2), and stored at -80°C. Prior to testing, the samples were ice-redissolved and gently blended to collect the supernatant. Following this, HDAC3 and FOXO1 were detected according to the operation steps as per the kit instructions. Meanwhile, each well's optical density (OD) was read with a microplate reader at a 450 nm detection wavelength (570 nm reference). During preliminary testing, standard solutions underwent twofold dilution series, and OD readings were used for standard curve plotting (4PL model; R-squared threshold: 0.99). The limit of detection (LOD) was determined as the concentration corresponding to the mean blank OD + 2SD. During cross-reactivity assessment, interfering proteins (e.g., IL-6, TNF-α at 100 ng/mL) were used, with acceptability thresholds set at <5%. Intra-assay coefficient of variation (CV) was evaluated by testing samples with known concentrations (high, medium, and low) in the same batch (CV ≤10%), while inter-assay CV was evaluated across different kit batches (CV ≤15%).

### Statistical methods

Statistical processing utilized SPSS 25.0. Categorical variables are presented as frequency counts with percentages [n(%)] and were analyzed using χ^2^ tests. The Shapiro-Wilk test was used to test for normal distribution. Continuous variables following normal distribution appeared as means with standard deviations, compared via independent t-tests. For skewed continuous data, we reported medians and interquartile ranges [Q (IQR)], employing Mann-Whitney U tests for comparisons. Longitudinal comparisons used repeated-measures ANOVA with Bonferroni-adjusted post-hoc tests. Effect sizes (Cohen's d) were calculated for significant differences. The combined diagnostic formula was derived via binary logistic regressionwith HDAC3/FOXO1 as covariates, optimized by maximum likelihood estimation. A *P*-value below 0.05 indicated statistical significance.

## Results

### Baseline data of the subjects were collected

No notable differences were found in baseline characteristics such as age, gender, or disease progression (*P*>0.05). Not only that, there was no significant difference in blood and Hcy test results between the two groups (*P*>0.05, Table 1).

**Table 1:** Clinical data of the CI and non-CI groups. Note: cognitive impairment (CI), cerebral stroke (CS), body mass index (BMI), recombinant tissue plasminogen activator (rt-PA), total cholesterol (TC), triglyceride (TG), low-density lipoprotein cholesterol (LDL-C), high-density lipoprotein cholesterol (HDL-C), homocysteine (Hcy).

Groups	Non-CI	CI	Statistics	P
	n=74	n=46		
Age	64.85±5.13	63.28±4.76	t=1.674	0.097
Gender			χ^2^=0.197	0.657
male	42 (56.76)	28 (60.87)		
female	32 (43.24)	18 (39.13)		
Smoking			χ^2^=0.441	0.507
yes	40 (54.05)	22 (47.83)		
no	34 (45.95)	24 (52.17)		
Drinking			χ^2^=0.288	0.592
yes	31 (41.89)	17 (36.96)		
no	43 (58.11)	29 (63.04)		
BMI (kg/m^2^)	25.58±2.88	25.39±2.21	t=0.389	0.698
Time from onset to admission (h)	13.35±5.27	13.17±5.79	t=0.173	0.863
High blood pressure			χ^2^=0.197	0.657
yes	42 (56.76)	28 (60.87)		
no	32 (43.24)	18 (39.13)		
Diabetes mellitus			χ^2^=0.167	0.683
yes	35 (47.30)	20 (43.48)		
no	39 (52.70)	26 (56.52)		
History of CS			χ^2^=0.137	0.711
yes	8 (10.81)	6 (13.04)		
no	66 (89.19)	40 (86.96)		
Options for treatment			χ^2^=0.592	0.442
rt-PA	55 (74.32)	37 (80.43)		
endovascular thrombectomy was<br>performed	19 (25.68)	9 (19.57)		
Types of CS			χ^2^=0.913	0.339
ischemia	67 (90.54)	39 (84.78)		
hemorrhagic	7 (9.46)	7 (15.22)		
TC (mmol/L)	5.92±0.59	6.03±0.61	t=0.973	0.332
TG (mmol/L)	1.88±0.36	1.92±0.36	t=0.609	0.544
LDL-C (mmol/L)	3.22±0.38	3.28±0.44	t=0.709	0.480
HDL-C (mmol/L)	1.15±0.21	1.17±0.19	t=0.514	0.608
Hcy (μmol/L)	15.78±3.77	16.29±3.35	t=0.745	0.458

### Dynamic changes in HDAC3 and FOXO1 in CS patients

The inter-group comparison of HDAC3 and FOXO1 levels revealed higher HDAC3 in CI group versus non-CI group across all timelines (T0, T1, T2; *P*<0.05), with the difference peakedat T2 (t=7.222, 7.348). As for FOXO1 expression, the two groups differed little at T0 (*P*>0.05), while higher levels were found in CI patients compared to non-CI cases at T1 and T2 (*P*<0.05). In-group comparisons showed barely changed HDAC3 and FOXO1 at T1 and T2 in non-CI group (*P*>0.05), lower versus T0 measurements (*P*<0.05). In CI patients, however, HDAC3 and FOXO1 began to decrease at T1 and further decreased at T2 (*P*<0.05, Table 2).

**Table 2:** Dynamic comparison of HDAC3 and FOXO1. Note: * means P < 0.05 compared with T0 and # means P < 0.05 compared with T1. histone deacetylase 3 (HDAC3), forkhead box protein O1 (FOXO1).

Groups	HDAC3 (ng/mL)					
	n	T0	T1	T2	F	P
Non-CI	74	4.59±0.83	4.15±0.72^*^	3.38±0.72^*#^	48.731	<0.001
CI	46	5.44±0.68	5.02±0.62^*^	4.29±0.59^*#^	39.524	<0.001
t		5.818	6.786	7.222		
*P*		<0.001	<0.001	<0.001		
	FOXO1 (pg/mL)					
	n	T0	T1	T2	F	P
Non-CI	74	76.86±6.80	63.37±8.01^*^	55.29±7.37^*#^	160.207	<0.001
CI	46	77.10±7.83	70.30±5.99^*^	66.11±8.57^*#^	24.883	<0.001
t		0.176	5.053	7.348		
P		0.861	<0.001	<0.001		

### Evaluation effects of HDAC3 and FOXO1 on CI

**Figure 2:**
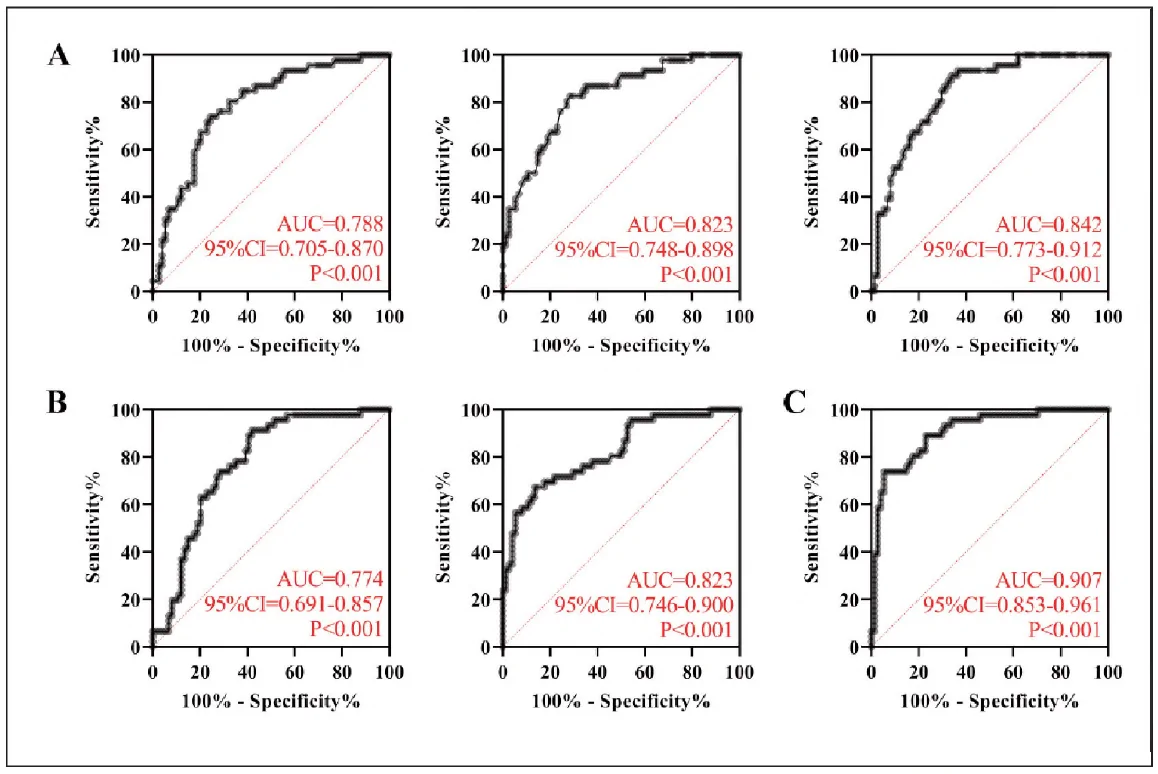
Diagnostic value of HDAC3 and FOXO1 for CI. A: The diagnostic effect of HDAC3 at T0, T1 and T2 on CI in CS patients. B: The diagnostic effect of FOXO1 at T0, T1 and T2 on CI in CS patients. C: Effect of HDAC3 and FOXO1 combined in the diagnosis of CS at T2.

Diagnostic analysis using HDAC3 and FOXO1 levels at T0, T1, and T2 timepoints revealed that HDAC3 exhibited consistent predictive value for CI occurrence in CS patients at all measured intervals. The highest diagnostic accuracy was observed at T2 (AUC = 0.842). In contrast, FOXO1 levels at T0 showed no significant difference between CI and non-CI groups, precluding its use for early assessment. However, FOXO1 demonstrated clinically meaningful predictive capability at later stages (T1 and T2), with superior performance at T2 (AUC = 0.823). According to the diagnostic effect of HDAC3 and FOXO1 at T2, we further established a diagnostic scheme for combined detection of HDAC3 and FOXO1 [Diagnostic_(combination)_ =-17.323 + 1.793x HDACA+0.165xFOXO1]. ROC curve analysis showed that the combination of HDAC3 and FOXO1 at T2 increased the AUC of CI to 0.907, and the specificity and sensitivity reached 73.91% and 94.59% (*P*<0.001, Figure 2 and Table 3).

**Table 3:** Diagnostic value of HDAC3 and FOXO1 for CI.

		Youden index_(max)_	Cut-off	Sensitivity (%)	Specificity (%)
T0	HDAC3	49.59	>5.13 ng/mL	73.91	75.68
	FOXO1	-	-	-	-
T1	HDAC3	54.23	>4.54 ng/mL	82.61	71.62
	FOXO1	49.41	>64.28 pg/mL	91.30	58.11
T2	HDAC3	57.52	>3.51 ng/mL	91.30	66.22
	FOXO1	53.88	>61.53 pg/mL	67.39	86.49
Diagnostic_(combination)_		68.51	>0.57	73.91	94.59

### Association of HDAC3, FOXO1 and CS prognostic recurrence risk

Follow-up results showed that 32 patients had CS recurrence. The comparison results showed that HDAC3 and FOXO1 in patients with recurrence were higher than those in patients without recurrence at T0-T2 (*P*<0.05). Among them, the difference between the two groups at T2 was also the most significant (*P*<0.001, Table 4).

**Table 4:** Comparison of HDAC3 and FOXO1 between patients with recurrent prognosis and those without recurrence. Note: * means P < 0.05 compared with T0 and # means P < 0.05 compared with T1.

Groups	HDAC3 (ng/mL)					
	n	T0	T1	T2	F	*P*
No recurrence	88	4.79±0.83	4.36±0.78^*^	3.52±0.73^*#^	12.021	<0.001
Recurrence	32	5.28±0.92	4.84±0.76^*^	4.31±0.68^*#^	60.023	<0.001
t		2.813	2.967	5.341		
*P*		0.006	0.004	<0.001		
Groups	FOXO1 (pg/mL)					
	n	T0	T1	T2	F	*P*
No recurrence	88	76.11±6.96	64.99±7.44^*^	56.38±7.86^*#^	18.843	<0.001
Recurrence	32	79.27±7.38	68.88±8.97^*^	67.86±8.27^*^	156.124	<0.001
t		2.163	2.395	6.976		
*P*		0.033	0.018	<0.001		

### The evaluation effect of HDAC3 and FOXO1 on the prognosis and recurrence of CS

**Figure 3:**
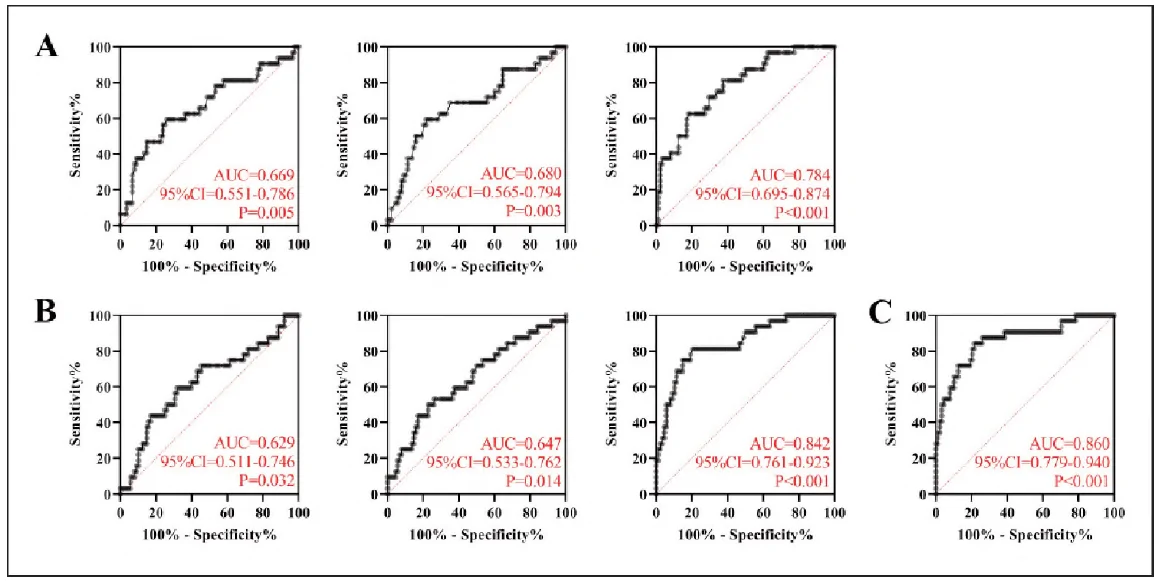
Diagnostic value of HDAC3 and FOXO1 for prognostic recurrence risk in CS. A: Diagnostic role of HDAC3 in the prognostic recurrence risk of CS at T0, T1, and T2. B: Diagnostic role of FOXO1 in the prognostic recurrence risk of CS at T0, T1, and T2. C: Effect of HDAC3 and FOXO1 combined in the diagnosis of prognostic recurrence risk of CS at T2.

According to the detection results of HDAC3 and FOXO1 at T2, a combined prediction formula for CS prognosis and recurrence was established: Diagnostic_(combination)_ =-13.634+0.925xHDACA+ 0.145xFOxO1, and then ROC curve analysis was performed. The results showed that the combined diagnosis of HDAC3 and FOXO1 had a sensitivity of 84.38% and a specificity of 78.41% in predicting the recurrence of CS within 1 year, and its AUC (0.860) was also significantly higher than that of HDAC3 and FOXO1 alone, suggesting that the two groups had excellent prognostic evaluation effect on CS (Figure 3 and Table 5).

**Table 5:** Diagnostic efficacy of HDAC3 and FOXO1 for prognostic recurrence risk in CS.

		Youden index_(max)_	Cut-off	Sensitivity (%)	Specificity (%)
T0	HDAC3	33.24	>5.34 ng/mL	59.38	73.86
	FOXO1	27.56	>78.66 pg/mL	59.38	68.18
T1	HDAC3	37.78	>4.90 ng/mL	59.38	78.41
	FOXO1	27.27	>69.23 pg/mL	50.00	77.27
T2	HDAC3	44.32	>4.23 ng/mL	62.50	81.82
	FOXO1	60.80	>61.09 pg/mL	81.25	79.55
Diagnostic_(combination)_		62.78	>0.23	84.38	78.41

## Discussion

Post-CS CI is the core complication that affects patients' quality of life, highlighting the clinical implications of early CI identification and intervention for prognosis enhancement [11]. However, traditional neuropsychological scales and imaging examinations demonstrate limitations like insufficient timeliness and strong subjectivity, which make it difficult to meet the needs of rapid and accurate diagnosis and treatment in emergency situations. As we all know, it is generally believed that blood lipid is the main cause of cerebrovascular obstruction in the clinic, and it is also the key observation index of CS progression [12]. Hcy is one of the most classical independent risk factors for CS [13]. However, when comparing the baseline data of the two groups of subjects, we found that there were no differences in the levels of blood lipids (TC, TG, LDL-C, HDL-C) and Hcy between the CI group and the non-CI group. It can be seen that although these indicators have been repeatedly confirmed to be related to CS [14][15], it is still difficult to evaluate the occurrence of CI by blood lipids and Hcy. This once again emphasizes the importance of finding new indicators for disease assessment.

In this study, T0 (baseline), T1 (2h post-reperfusion: acute oxidative stress window), and T2 (24h: early neural repair phase) were selected to capture dynamic biomarker transitions. The results of this study showed that HDAC3 was higher in CI cases than in non-CI patients at T0, T1, and T2 (P<0.05), while FOXO1 exhibited more obvious differences at T1 and T2. Longitudinal monitoring revealed that biomarker levels in the non-CI group showed progressive decline with treatment duration, whereas the CI group demonstrated smaller reductions with occasional fluctuations. This pattern suggests that distinct mechanistic pathways may underlie their respective roles in cognitive deterioration.

HDAC3, as a key molecule of epigenetic regulation, can modify histones and non-histones through deacetylation, inhibit synaptic plasticity-associated gene levels, and promote the transcription activation of inflammatory factors and apoptosis genes, which is an important driving factor of nerve injury [16]. Animal experiments by Fang H et al. [17] showed a marked increase in HDAC3 nuclear translocation following ischemic brain injury, inducing synaptic dysfunction and cognitive defects. HDAC inhibitors can improve spatial memory and learning ability by reversing chromatin remodeling [18]. In this study, we found that HDAC3 increased evidently at T0 while correlating positively with NIHSS scores and negatively with MoCA values. It suggests that HDAC3 might accelerate neuronal death by amplifying postischemia inflammatory cascades. It is worth noting that in the CI group, HDAC3 remained at a high level at T2, indicating a potential close relationship between its sustained high expression and obstructed nerve repair and poor long-term cognitive outcomes. In contrast, FOXO1, as the core downstream factor of the insulin/PI3K/Akt pathway, influences neuron survival by mediating oxidative stress and regulating mitochondrial function [19]. In the early stage of ischemia, Akt activity declines lead to FOXO1 nucleus translocation, activating antioxidant genes and proapoptosis genes [20]; reperfusion reactivates Akt, and phosphorylates FOXO1 to promote its degradation, thus reducing oxidative damage [21]. In this study, the rapid decrease in FOXO1 at T1, as well as its strong correlation with NIHSS and MoCA, indicates the potential ability of FOXO1 down-regulation to predict the improvement of neuron survival status and cognitive function recovery. Of note, despite the overall reduction of FOXO1 inCI group following treatment, intermittent fluctuations were observed in a subset of patients, potentially attributable to reperfusion injury or incomplete resolution of inflammation. This implies that FOXO1 variability may indicate the equilibrium between oxidative stress and recovery processes.

Subsequently, we analyzed the evaluation effects of HDAC3 and FOXO1 at different time points. At T0, only HDAC3 exhibited certain diagnostic value for CI, but with limited diagnostic specificity (75.68%), possibly due to the influence of many confounding factors (e.g., infarction site, collateral circulation) on the degree of baseline injury. Nevertheless, HDAC3, as an early warning marker, can still provide a basis for high-risk stratification. At T1, HDAC3 showed a milder magnitude of reduction in CI group compared to non-CI group, suggesting that its dynamic changes can reflect reperfusion therapy efficacy. However, FOXO1 in CI patients rapidly decreased to the level of non-CI group, indicating its utility as a biomarker of successful reperfusion. Given their respective capacities to elicit rapid therapeutic responses, both demonstrate predictive potential for early cognitive recovery. At T2, due to the obstruction of nerve repair in CI group, HDAC3 maintained a high level, while FOXO1 fluctuated. Consequently, the contrast between CI and non-CI groups reaches its peak, highlighting HDAC3 and FOXO1 as highly effective diagnostic markers for CI. The combined detection of HDAC3 and FOXO1 can improve the AUC of CI in CS patients to 0.907, which has extremely high reference value. These findings further suggest that biomarker levels during the neural repair homeostasis stage are more indicative of long-term cognitive outcomes.

In addition to early CI assessment, this study reveals for the first time a significant association of HDAC3/FOXO1 dynamic changes with long-term recurrence risk in CS. The serum levels of HDAC3 and FOXO1 in the recurrence group were significantly higher than those in the non-recurrence group at T0-T2, and the difference between the two groups was the most significant at T2. This persistent high expression pattern suggests that persistent dysregulation of the HDAC3/FOXO1 axis is not only a marker of acute neurological injury and CI (as discussed above), but also may reflect vascular endothelial dysfunction or the pathological microenvironment of unstable atherosclerotic plaques, providing a biological basis for recurrent CS. Based on the detection values of HDAC3 and FOXO1 at T2, a recurrence risk prediction formula was established. The AUC of the combined model for predicting CS recurrence within 1 year was 0.860, which was significantly better than that of a single biomarker. The reason may be that the sustained high expression of HDAC3 promotes the transcription of pro-inflammatory factors through epigenetic regulation, exacerbates the inflammatory response of vascular endothelium, and thus increases the risk of thrombosis [22]. The abnormal level of FOXO1 is directly related to the imbalance of oxidative stress, and its nuclear translocation can activate the pro-apoptotic pathway, leading to the dysfunction of vascular smooth muscle cells and accelerating the progression of atherosclerosis [23]. The above processes together constitute the molecular basis of CS recurrence, and are closely related to the degree of neurological deficit.

Based on the results of this paper, HDAC3 >3.51 ng/mL + FOXO1 >61.53 pg/mL at T2 prediets CI risk (AUC = 0.907). In the future, both HDAC3 and FOXO1 have a certain application potential in the evaluation of emergency triage and rehabilitation efficacy. Although statistical efficacy was demonstrated through G-Power estimation, verification across multiple centers with expanded sample sizes is required to address potential confounding from regional or intervention disparities. Besides, we did not investigate HDAC3 and FOXO1 dynamics during the hyperacute phase (<4.5 hours) or late stage (>24 hours), potentially missing critical pathological transitions, limiting the understanding of the dynamics of the initial biomarkers. Similarly, HDAC3 and FOXO1 expression may be interfered by other pathological processes, necessitating further validation through proteomics or genetic knockout models to confirm specificity. Future studies should also employ basic experiments to further explore the direct mechanism and related pathways of HDAC3 and FOXO1 in nerve repair, so as to provide more comprehensive references and clinical guidance.

## Conclusion

HDAC3 levels correlate with acute-phase neural damage severity, whereas FOXO1 serves as a marker for post-reperfusion oxidative stress mitigation. Both of them can provide a new clinical diagnostic scheme for early identification of high-risk CI patients and prognosis of CS recurrence.

## Dodatak

### Consent to publish

All authors gave final approval of the version to be published.

### Availability of data and materials

The data used to support the findings of this study are available from the corresponding author upon request.

### Funding

This study was supported by the Xinjiang Uygur Autonomous Region Traditional Chinese Medicine Youth Science and Technology Talent Project (No. ZYQ2025013); In-hospital Project of Changji Branch of the First Affiliated Hospital of Xinjiang Medical University (No. 202204).

### Acknowledgements

Not applicable.

### Author contributions

Yue Dou conceived and designed the study, Xintong Li and Lulu Wang wrote and revised the manuscript, Bin Xiao collected and analyzed data, Zhengkun Liu supervised the study, Xintong Li and Lulu Wang made equal contributions in this work as co-first authors. All authors read and approved the final submitted manuscript.

### Conflict of interest statement

All the authors declare that they have no conflict of interest in this work.
